# Nonadiabatic dynamics: The SHARC approach

**DOI:** 10.1002/wcms.1370

**Published:** 2018-05-09

**Authors:** Sebastian Mai, Philipp Marquetand, Leticia González

**Affiliations:** ^1^ Institute of Theoretical Chemistry, Faculty of Chemistry University of Vienna Vienna Austria

**Keywords:** Ab initio molecular dynamics, excited states, nonadiabatic dynamics, surface hopping, SHARC

## Abstract

We review the Surface Hopping including ARbitrary Couplings (SHARC) approach for excited‐state nonadiabatic dynamics simulations. As a generalization of the popular surface hopping method, SHARC allows simulating the full‐dimensional dynamics of molecules including any type of coupling terms beyond nonadiabatic couplings. Examples of these arbitrary couplings include spin–orbit couplings or dipole moment–laser field couplings, such that SHARC can describe ultrafast internal conversion, intersystem crossing, and radiative processes. The key step of the SHARC approach consists of a diagonalization of the Hamiltonian including these couplings, such that the nuclear dynamics is carried out on potential energy surfaces including the effects of the couplings—this is critical in any applications considering, for example, transition metal complexes or strong laser fields. We also give an overview over the new SHARC2.0 dynamics software package, released under the GNU General Public License, which implements the SHARC approach and several analysis tools. The review closes with a brief survey of applications where SHARC was employed to study the nonadiabatic dynamics of a wide range of molecular systems.

This article is categorized under:

Theoretical and Physical Chemistry > Reaction Dynamics and Kinetics
Software > Simulation Methods
Software > Quantum Chemistry

## 1 INTRODUCTION

Nonadiabatic dynamics in molecules involves processes in which the nuclear motion is affected by more than one electronic state. These processes can, for example, take place when a molecule is irradiated by light. In such a situation, nuclear motion cannot be described anymore in the frame of the Born–Oppenheimer approximation, which assumes that only one electronic state affects the nuclei. Instead, whenever two or more electronic states have similar energies and state‐to‐state couplings are sufficiently large, population transfer from one state to another will take place.

Depending on the type of electronic states involved and the type of the state‐to‐state couplings, nonadiabatic processes can be classified into internal conversion (IC) and intersystem crossing (ISC). In IC, states of the same spin multiplicity (e.g., two singlet states) interact with each other via the so‐called nonadiabatic couplings (NACs). In ISC, states of different spin multiplicity (e.g., a singlet and a triplet) interact via the relativistic spin–orbit couplings (SOCs), while in the frame of nonrelativistic quantum chemistry, ISC is naturally forbidden by spin symmetry.

Nonadiabatic phenomena are relevant in many photophysical and photochemical processes, including some fundamental biochemical phenomena such as visual perception, photosynthesis, bioluminescence, DNA photodamage and repair, or vitamin D synthesis. Therefore, a number of computational methods have been developed in the last decades to simulate nonadiabatic dynamics, with trajectory surface hopping (SH) being one of the most popular (Barbatti, 2011).

In this work, we focus on a generalized version of trajectory SH, coined SHARC (Surface Hopping including ARbitrary Couplings), as it can deal with any type of couplings on the same footing, for example, NACs and SOCs. SHARC was first developed in 2011 with the aim to perform nonadiabatic dynamics simulations including SOCs and field‐matter interactions in systems with many degrees of freedom (Bajo et al., 2012; Richter, Marquetand, González‐Vázquez, Sola, & González, 2011; Richter, Marquetand, González‐Vázquez, Sola, & González, 2012a). However, SHARC can be also used in a general way to study IC only—either within singlet states, or within states of other multiplicities, for example, triplets, in which case also SOCs are required. In 2014, after a major overhaul, the first version of the SHARC dynamics suite was made publicly available (Mai et al., 2014); details of the early software implementations have been reported elsewhere (Mai, Marquetand, & González, 2015; Mai, Plasser, Marquetand, & González, 2018; Mai, Richter, Marquetand, & González, 2015b). Here, we present an up‐to‐date overview over the SHARC approach and its newest implementation, the new dynamics suite SHARC2.0 (Mai et al., 2018) and its capabilities, as well as a brief survey of some of the applications that the excited‐state dynamics community carried out using SHARC. In the following, we first briefly describe the main ideas behind SH, before introducing the particularities of the SHARC approach.

## 2 THE SH METHOD

### 2.1 What is surface hopping?

A full‐dimensional quantum mechanical treatment of the nuclear motion of a large, polyatomic molecule is nowadays unfeasible due to the exponential scaling of the computational effort with the number of dimensions—this is called the dimensionality bottleneck of quantum mechanical methods (Meyer, Gatti, & Worth, 2009). This bottleneck spurred the development of mixed quantum‐classical methods that only treat the electrons quantum mechanically, while the nuclear motion is treated classically (Doltsinis, 2006; Marx & Hutter, 2000; Tully, 1998). Among them, SH (Barbatti, 2011; Doltsinis, 2006; Subotnik et al., 2016; Tully, 1990; Tully & Preston, 1971; L. Wang, Akimov, & Prezhdo, 2016) is probably one of the most prominent methods, and it is the basis for SHARC. The advantages of SH (Barbatti, 2011)—which are the main reason for its popularity—are simplicity (which aids both development of SH methods and interpretation of results), practicality (allowing on‐the‐fly implementations and trivial parallelization), and the ability to include all nuclear degrees of freedom at feasible computational cost. The disadvantage of SH (Barbatti, 2011; Tully, 1990) is that it naturally misses truly quantum effects, such as a correct description of the zero‐point energy, tunneling, or nuclear interferences.

In SH, the quantum and the classical parts are described as follows. The electrons are represented by a time‐dependent electronic wave function |Φ^el^(*t*)〉, written as a linear combination of electronic basis states

(1) $$|\Phi^{\mathrm{el}}\left(t\right)〉=\sum_{\alpha }c_{\alpha }\left(t\right)|\Psi_{\alpha }\left(t\right)〉$$

where *α* runs over all basis states, *c*_*α*_(*t*) are the time‐dependent coefficients, and |Ψ_*α*_(*t*)〉 are the basis states. As the choice of the set of these basis states is not irrelevant, it will be discussed in a separate section below. The temporal evolution of this wave function follows the time‐dependent Schrödinger equation, and is affected by the classical nuclear coordinates **R**(*t*) through the parametric dependence of the electronic Hamiltonian on the vector **R**(*t*). Additionally, each nucleus *A* obeys the classical equation of motion

(2) $$M_{A}\frac{\partial^{2}R_{A}}{\partial t^{2}}=-\frac{\partial E^{\mathrm{el}}}{\partial R_{A}}$$

where the classical force on nucleus *A* is the negative gradient of the electronic energy. In this way, the nuclei follow classical *trajectories* (defined by the positions **R** of all nuclei changing in time), which are influenced by the quantum‐mechanically treated electrons. As can be seen, the classical nuclear evolution and the quantum‐mechanical electronic evolution are intimately coupled.

Unlike a quantum wave packet, the classical nuclei can only follow one particular force in each instant of time, and in SH, this force is given by the gradient of the *active* electronic state. In order to determine the active state, there exists quite a large number of prescriptions, which give rise to many different SH variants (e.g., the works of Herman, 2005; Jasper, Stechmann, & Truhlar, 2002; Martens, 2016; Nangia, Jasper, Miller, & Truhlar, 2004; Oloyede, Milnikov, & Nakamura, 2006; L. Wang et al., 2016; Webster, Wang, Rossky, & Friesner, 1994). As reviewing all these variants is beyond the scope of this work, here we focus on the idea of “fewest switches” SH (Hammes‐Schiffer & Tully, 1994; Tully, 1990), where the composition of the time‐dependent electronic wave function is monitored through the population in each electronic state, |*c*_*α*_(*t*)|^2^. When the population of the current active state decreases (and only then according to the fewest switches criterion), one computes the probabilities to switch the active state to any other state. Based on such probabilities, a stochastic algorithm chooses the new active state (Tully, 1990). If the active state is changed, a so‐called surface hop is performed, which gave the method its name. From this instant of time, the trajectory continues on the new active state, possibly hopping again at a later time.

### 2.2 Additional ingredients of surface hopping

Besides the main idea described above, several additional aspects are relevant in SH simulations.

#### 2.2.1 Kinetic energy adjustment

As the new active state after a hop will likely have a potential energy different from the former one, it is necessary to adjust the kinetic energy of the system so that the total energy is conserved during a hop. The original approach (Tully, 1990; Tully & Preston, 1971) is to rescale the component of the velocity vector parallel to the NAC vector between old and new active state. This approach is rigorous (Coker & Xiao, 1995; Hack, Jasper, Volobuev, Schwenke, & Truhlar, 1999; Herman, 1984) and size‐consistent, but requires the computation of NAC vectors. If these are not available, the usual approach is to rescale the whole velocity vector **v** (Fabiano, Keal, & Thiel, 2008; Müller & Stock, 1997; Tapavicza, Tavernelli, & Rothlisberger, 2007), although Hack et al. (1999) also suggested rescaling along the gradient difference vector. It can also happen that the kinetic energy cannot be adjusted such that the total energy remains conserved, for example, because the kinetic energy available along the NAC vector is smaller than the potential energy difference in the hop. This is called a “frustrated” hop, which is rejected such that the active state does not change. However, some authors (Hammes‐Schiffer & Tully, 1994) suggest to invert the component of the velocity vector parallel to the relevant NAC vector whenever a frustrated hop occurs.

The situation is slightly more complicated in the presence of a laser field, because in this case, the Hamiltonian is time‐dependent and thus the total energy is not necessarily conserved. In such a case, the algorithm needs to distinguish between nuclear‐motion‐induced hops and laser‐induced hops (or needs to interpolate between these two limiting cases). A simple approach to this is to check whether the energy change during the hop is compatible with the laser frequency (Mai, Richter, Heindl, et al., 2018). Alternatively, one can inspect the origin of the Hamiltonian matrix elements which induced the hop (Bajo, Granucci, & Persico, 2014; Thachuk, Ivanov, & Wardlaw, 1998).

#### 2.2.2 Decoherence

In the evolution of the coefficients *c*_*α*_(*t*) (which will be discussed in more detail below) in SH, an artificial system with complete coherence is assumed (Granucci, Persico, & Zoccante, 2010; Jaeger, Fischer, & Prezhdo, 2012; Subotnik et al., 2016; Subotnik, Ouyang, & Landry, 2013). This means that for each trajectory, the electronic population situated on all states follows the gradient of the active state, whereas in quantum mechanics, the population of each state follows the gradient of its respective state. As a consequence, SH is usually overcoherent and a decoherence correction scheme needs to be applied to obtain reasonable results. These correction schemes typically reduce or collapse the population of the inactive states according to an estimate of the quantum‐mechanical decoherence rate. The decoherence rate is usually estimated according to some semiclassical approximation, for example, based on phenomenological arguments (Granucci & Persico, 2007; Zhu, Nangia, Jasper, & Truhlar, 2004) or based on the assumption of frozen Gaussians which move apart because the different states have different energies (Granucci et al., 2010) or different gradients (Jain, Alguire, & Subotnik, 2016).

#### 2.2.3 Ensemble of trajectories

Because of the stochastic process involved in hopping, and also because a single classical trajectory cannot reproduce the branching of a wave packet into different reaction channels, SH requires an ensemble of trajectories, each starting from a different initial condition (Tully, 1990). The size of the ensemble should be large enough so that the initial wave packet (e.g., the vibrational ground state of the electronic ground state) is well characterized. Depending on the excitation procedure and the density of electronic excited states, it might also be necessary to consider in the ensemble a distribution over different initial states, such that the excitation process is well described (Barbatti, 2011). Due to the stochastic nature of the hopping procedure, in principle, one should also run multiple trajectories with identical initial conditions and different random number sequences, such that the encountered hopping situations are well sampled (Barbatti, 2011; Tully, 1990). In practice, however, to keep computational cost manageable, the actual number of trajectories is usually restricted; particularly, if the underlying on‐the‐fly method (see below) is computationally demanding. Even at the expense of statistical significance, most often quality is preferred to quantity: few trajectories with rather expensive but accurate potentials are preferable to many trajectories with cheap but unrealistic potentials.

#### 2.2.4 Generation of initial conditions

As hinted above, SH simulations require to define initial conditions, in terms of initial positions **R**(0), initial velocities **v**(0), and initial electronic states. Most often, generating initial conditions involves first sampling a large set of (**R**(0), **v**(0)) pairs from the relevant vibrational state in the electronic ground state. In practice, this involves either the computation of a Wigner distribution of a harmonic oscillator model around the ground state minimum, or carrying out a molecular dynamics simulation on the ground state potential energy surface (PES). After (**R**(0), **v**(0)) pairs are found, the initial electronic state is defined based on vertical excitation energies and oscillator strength as well as some assumptions about the excitation process.

#### 2.2.5 Choice of the electronic structure method

During a SH simulation, several electronic quantities are required at every simulation time step: energies, gradients, state‐to‐state couplings, (transition) dipole moments, and so on. In most cases, these quantities are directly computed along the simulation, which is often referred to as “on‐the‐fly” or “direct” dynamics (Helgaker, Uggerud, & Jensen, 1990). The quantities can be obtained with any electronic structure method that provides electronic excited states as well as their gradients and couplings. Those include many ab initio and density functional theory methods, but semiempirical methods (Thiel, 2014) or density functional tight binding (DFTB; Seifert & Joswig, 2012) can also be used. For large systems that can be partitioned, hybrid quantum mechanics/molecular mechanics (QM/MM) methods (Senn & Thiel, 2009) could also be applied. Alternatively, instead of relying on on‐the‐fly calculations, it is also possible to employ analytical model functions to describe the required quantities. Naturally, the method chosen affects the shape of the PESs and thus has a large impact on the reliability of the dynamical results and its interpretation. The electronic structure methods available within the SHARC2.0 package will be discussed below.

## 3 THE SHARC APPROACH

### 3.1 Basis functions representations

The SHARC approach is a generalization of the SH method to the case of any arbitrary state‐to‐state couplings. The description of these couplings strongly depends on the definition of the electronic Hamiltonian, as well as on the choice of the basis functions |Ψ_*α*_(*t*)〉 for the electronic wave function |Φ^el^(*t*)〉, cf. Equation (1). Therefore, in the following, we will briefly introduce the relevant theory and show how this choice affects the basic equations of SH within SHARC.

The standard electronic Hamiltonian in most quantum chemistry calculations is the molecular Coulomb Hamiltonian (MCH), which can be written (in a.u.) as:

(3) $${\hat{H}}^{\mathrm{MCH}}=-\sum_{i}\frac{1}{2}\nabla_{i}^{2}+\sum_{A<B}\frac{Z_{A}Z_{B}}{|R_{A}-R_{B}|}-\sum_{A}\sum_{i}\frac{Z_{A}}{|R_{A}-r_{i}|}+\sum_{i<j}\frac{1}{|r_{i}-r_{j}|}$$

As the name suggests, this definition of the Hamiltonian consider neither fields external to the molecule nor any interactions beyond the Coulomb one. In order to incorporate arbitrary couplings into SH, one needs to extend this restrictive Hamiltonian, leading to the total Hamiltonian:

(4) $${\hat{H}}^{\text{total}}={\hat{H}}^{\mathrm{MCH}}+{\hat{H}}^{\text{additional}}$$

Note that with ${\hat{H}}^{\text{total}}$, we refer to the total *electronic* Hamiltonian, since the nuclear kinetic energy is always treated classically in SH. An example for a term in ${\hat{H}}^{\text{additional}}$ is SOC, which is described by the Breit–Pauli Hamiltonian (Marian, 2012; Pauli Jr., 1927)—or the different mean‐field approximations (Heß, Marian, Wahlgren, & Gropen, 1996; Marian, 2012; Neese, 2005) to it—and is necessary to simulate ISC. Another additional term could be the electric field–dipole moment coupling, which is necessary to describe light‐matter interactions and thus to simulate explicitly all light‐induced processes, such as excitation, stimulated emission, or Stark effects (Marquetand, Richter, González‐Vázquez, Sola, & González, 2011; Sanz‐Sanz, Richings, & Worth, 2011; Sussman, Townsend, Ivanov, & Stolow, 2006).

With the definition of the electronic wave function in Equation (1) and the electronic Hamiltonian in Equation (4) at hand, one can now introduce the electronic time‐dependent Schrödinger equation (in a.u.):

(5) $${\hat{H}}^{\text{total}}|\Phi^{\mathrm{el}}\left(t\right)〉=i\frac{\partial }{\partial t}|\Phi^{\mathrm{el}}\left(t\right)〉$$

By inserting Equation (1) and left‐multiplying with 〈Ψ_*β*_(*t*)|, one can derive the equation of motion for the electronic wave function coefficients (Doltsinis, 2006):

(6) $$\frac{\partial }{\partial t}c_{\beta }=-\sum_{\alpha }\left[iH_{\beta \alpha }+T_{\beta \alpha }\right]c_{\alpha }$$

where we used *δ*_*βα*_ = 〈Ψ_*β*_|Ψ_*α*_〉, $H_{\beta \alpha }=〈\Psi_{\beta }|{\hat{H}}^{\text{total}}|\Psi_{\alpha }〉$, and *T*_*βα*_ = 〈Ψ_*β*_|*∂*/*∂t*|Ψ_*α*_〉. The time‐derivative coupling *T*_*βα*_ is usually computed as *T*_*βα*_ = **v**·**K**_*βα*_, where **K**_*βα*_ = 〈Ψ_*β*_|*∂*/*∂* **R**|Ψ_*α*_〉 is the NAC vector.

All quantities in the equation of motion for the electronic propagation (6), as well as the electronic energy in Equation (2), depend on the choice of the set of electronic basis states {|Ψ_*α*_〉}. Within this work, we refer to these different possible choices of basis state sets as “representations.” As long as the basis set is complete, the choice of representation does not matter in a fully quantum‐mechanical calculation. However, in the case of classical dynamics and more specifically in SH, the choice of representation does matter. For example, the representation affects the form of the PESs, such that in one representation a classically forbidden barrier appears, whereas in another representation the barrier can be surmounted by the classical nuclei. Moreover, the representation affects how localized or delocalized the state‐to‐state couplings in **H** or in **K** (Equation (6)) are, which in turn affects the number of hops in SH. For ISC dynamics, it should also be noted that the representation influences the population transfer involving the different components of multiplets (Granucci, Persico, & Spighi, 2012).

In principle, different representations could be used in SH, and there is a significant body of literature on the topic. Already Tully (1998) stated that the adiabatic representation should be superior for SH, compared to any diabatic representation. Moreover, Subotnik et al. (2016, 2013) and Kapral (2016) showed that SH is related to the mixed quantum‐classical Liouville equation and (among other results) found that surface hops should optimally only occur in small regions of configuration space with large couplings. This means that the optimal basis for SH is the one where the state‐to‐state couplings are very localized. Abedi, Agostini, Suzuki, and Gross (2013) and Fiedlschuster, Handt, Gross, and Schmidt (2017) have shown that SH using the adiabatic representation reproduces very well the results of the exact factorization formalism, whereas other representations deliver unphysical results. This fully agrees with our earlier findings (Bajo et al., 2012), where different representations were compared in the presence of strong laser fields. Based on this body of literature, most authors agree that the best representation for SH should be the adiabatic basis, that is, the basis composed of the *eigenstates of the total electronic Hamiltonian*, as defined in Equation (4).

In principle, the eigenstates of the total electronic Hamiltonian should be obtained with quantum chemistry software. Hence, if we can use quantum chemistry software to compute the eigenstates of ${\hat{H}}^{\text{total}}$, together with energies, gradients, and NACs, we can apply the standard SH formalism without any modifications. However, most quantum chemistry software used nowadays is primarily intended to find eigenstates of the MCH. Considering the additional coupling terms in ${\hat{H}}^{\text{additional}}$ during wave function computation makes the quantum chemistry computations much more involved. For example, if ${\hat{H}}^{\text{additional}}={\hat{H}}^{\mathrm{SOC}}$ (e.g., the Breit–Pauli Hamiltonian or an effective one‐electron spin–orbit operator) one is in the realm of relativistic quantum chemistry, which is considerably more complicated than nonrelativistic quantum chemistry. This is due to the need for at least two‐component wave functions, significantly larger basis sets and configuration interaction (CI) expansions, and a larger number of states to compute (since multiplet components need to be converged separately). Furthermore, quantities such as NAC vectors are not routinely available from relativistic quantum chemistry. Alternatively, if ${\hat{H}}^{\text{additional}}$ includes electric field–dipole moment couplings, then the quantum chemistry calculation needs to include an electric field, which is relatively easy to do. However, since the electric field will vary quickly, it might be necessary to perform the quantum chemistry calculations using very short time steps. For example, at 400 nm, the electric field changes from zero to maximum amplitude in 0.25 fs, making time steps of 0.1 fs or smaller desirable and thus strongly increasing the computational effort.

### 3.2 The basic idea behind SHARC

As we have seen, we are faced with the predicament that in the praxis quantum chemistry does not deliver the eigenstates of the total electronic Hamiltonian and all related quantities. In order to circumvent this problem, it is possible to find approximate eigenstates of the total Hamiltonian by applying quasi‐degenerate perturbation theory (QDPT; Mai et al., 2014; Vallet, Maron, Teichteil, & Flament, 2000; F. Wang & Ziegler, 2005). In this approach, one computes first a suitable set of eigenstates of the MCH {|$\Psi_{\alpha }^{\mathrm{MCH}}$〉}, for example, the few lowest singlet and/or triplet states. In the following, we shall call this set of eigenstates of the MCH the “MCH representation.” Note that other authors refer to it as “adiabatic spin‐diabatic” (Granucci et al., 2012) or “field‐free” (Mitrić, Petersen, & Bonačıć‐Koutecký, 2009) representation. In the basis of these states, we calculate all matrix elements $H_{\beta \alpha }^{\mathrm{MCH}}=〈\Psi_{\beta }^{\mathrm{MCH}}|{\hat{H}}^{\text{total}}|\Psi_{\alpha }^{\mathrm{MCH}}〉$, which form the total Hamiltonian matrix in the MCH representation, **H** ^MCH^. It is then possible to diagonalize this matrix

(7) $$H^{\mathrm{diag}}=U^{†}H^{\mathrm{MCH}}U$$

to obtain approximate eigenenergies (the diagonal elements of **H** ^diag^) and eigenstates of the total Hamiltonian:

(8) $$|\Psi_{\beta }^{\mathrm{diag}}〉=\sum_{\alpha }|\Psi_{\alpha }^{\mathrm{MCH}}〉U_{\alpha \beta }$$

Here, we call the set of eigenstates of ${\hat{H}}^{\text{total}}$ the “diagonal representation,” with other authors referring to it as “spin‐adiabatic” representation (Granucci et al., 2012) or “field‐dressed” representation (Thachuk, Ivanov, & Wardlaw, 1996). The approximation inherent in this QDPT approach is that all couplings with higher MCH states than the ones computed are neglected.

In a nutshell, the basic paradigm of the SHARC approach is to perform SH on the approximate eigenstates obtained through applying QDPT to a set of MCH states. As a consequence, in general, in SHARC simulations two representations are of prime relevance—the one in which the quantum chemistry is executed (the MCH representation) and the one in which the nuclear propagation is carried out (the diagonal one).

Additional representations might be beneficial for the a posteriori analysis of the results. An example would be a “diabatic” or “crude adiabatic” basis (Domcke, Yarkony, & Köppel, 2004), where the electronic wave function of each state does not change along nuclear coordinates. Such a basis cannot be rigorously defined for a polyatomic molecule (Kendrick, Mead, & Truhlar, 2000; Yarkony, 2004) but experimental observables from spectroscopy are often discussed in terms of such states and hence transforming the results to such a basis can be advantageous for the interpretation of the simulations. Hence, in the context of SHARC simulations, we label this representation the “spectroscopic” representation.

**Figure 1:**
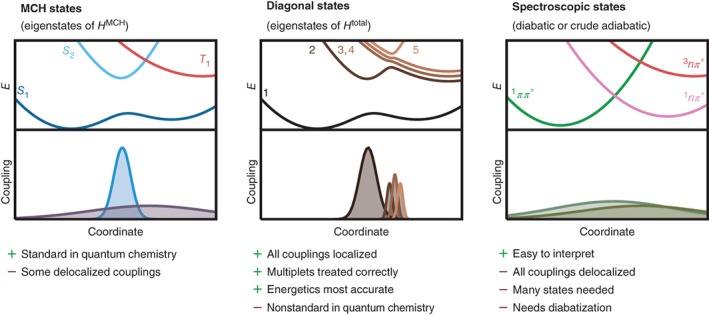
Schematic examples of potential energy surfaces (PESs) and coupling matrix elements in the three different representations discussed in the text (molecular Coulomb Hamiltonian [MCH], diagonal, and spectroscopic). The listed items below the figures summarize the advantages (+) and disadvantages (−) of these representations for SH simulations

The three types of representations mentioned above—the MCH, diagonal, and spectroscopic ones—are exemplified in Figure 1. In this example, in the MCH representation, two states of the same multiplicity (*S*_1_ and *S*_2_) form an avoided crossing, where localized NACs mediate population transfer, whereas states of different multiplicity (*S*_2_ and *T*_1_) can freely cross and are coupled by delocalized SOCs; multiplet components are exactly degenerate. In the diagonal representation, no states cross (although they might touch at a conical intersection), multiplet components are split up, and all couplings are localized. In contrast, in a diabatic representation, all states can freely cross, and all couplings are delocalized.

### 3.3 Practical implementations of the SHARC approach

In order to make the underlying idea of SHARC—SH on diagonal states obtained through QDPT—practical, several aspects of the SH algorithm needs to be adjusted to make it numerically stable and accurate.

First, it is necessary to propagate the electronic wave function coefficients **c** ^diag^(*t*) using only quantities in the MCH representation. The most straightforward procedure would be to apply the basis transformation analogous to Equation (7) to the equation of motion (6), which yields in matrix notation:

(9) $$\frac{\partial }{\partial t}c^{\mathrm{diag}}=-\left[iU^{†}H^{\mathrm{MCH}}U+v\cdot U^{†}K^{\mathrm{MCH}}U+U^{†}\frac{\partial U}{\partial t}\right]c^{\mathrm{diag}}$$

In order to propagate the coefficients from one time step *t* to the next *t* + Δ*t* step, this equation can be directly integrated by any suitable method, for example, Runge–Kutta/Butcher algorithms, or short‐time matrix exponentials.

However, the derivative **U** ^†^ *∂* **U**/*∂t* is numerically very difficult to handle because **U** is not uniquely determined by Equation (7) (see below) and because **U** might change very rapidly in the vicinity of near‐degeneracy points. This situation is not rare in ISC dynamics, because in all molecules there will be state pairs with small mutual SOCs, and whenever these states cross (a type of “trivial crossing”), the matrix **U** changes rapidly, which leads to a highly probable hop in the diagonal representation. Hence, it is advisable to exclude **U** ^†^ *∂* **U**/*∂t* from the integration of Equation (9). This can be achieved with the so‐called three‐step propagator approach (Mai, Marquetand, & González, 2015), where the computation of **c** ^diag^(*t* + Δ*t*) is split into three matrix–vector products:

(10) $$c^{\mathrm{diag}}\left(t+\Delta t\right)=U^{†}\left(t+\Delta t\right)P^{\mathrm{MCH}}\left(t+\Delta t,t\right)U\left(t\right)c^{\mathrm{diag}}\left(t\right)$$

As can be seen, this equation describes first a transformation from **c** ^diag^(*t*) to **c** ^MCH^(*t*), second a propagation from **c** ^MCH^(*t*) to **c** ^MCH^(*t* + Δ*t*), and third a transformation form **c** ^MCH^(*t* + Δ*t*) to **c** ^diag^(*t* + Δ*t*). Since the second step (multiplication by **P** ^MCH^(*t* + Δ*t*, *t*)) is the electronic propagation in the MCH basis and involves only MCH quantities, no **U** matrix is involved and numerical problems are avoided. The propagator matrix **P** ^MCH^(*t* + Δ*t*, *t*) can be obtained by integrating over **H** ^MCH^ and **v** ·**K** ^MCH^, for example, with Runge–Kutta/Butcher algorithms, or short‐time matrix exponentials (details and equations can be found in Mai, Marquetand, and González (2015) or in the SHARC manual as given in “Further Reading”). The advantage of using the three‐step propagator instead of a “one‐step” propagator (i.e., directly integrating Equation (9)) is that the coefficients can be smoothly propagated with relatively long time steps even in the presence of state crossings with very small off‐diagonal couplings (called “trivial crossings” by other authors (Fernandez‐Alberti, Roitberg, Nelson, & Tretiak, 2012; Granucci, Persico, & Toniolo, 2001; Plasser et al., 2012; L. Wang & Prezhdo, 2014)).

Instead of computing the propagator **P** ^MCH^(*t* + Δ*t*, *t*) from **H** ^MCH^ and **v**·**K** ^MCH^, it can be computed with the local diabatization procedure (Granucci et al., 2001). This procedure does not require NACs **K** ^MCH^ but instead employs the overlap matrix **S** ^MCH^ between states of subsequent time steps (i.e., at different geometries and with different orbitals), with elements:

(11) $$S_{\alpha \beta }^{\mathrm{MCH}}\left(t,t+\Delta t\right)=〈\Psi_{\alpha }^{\mathrm{MCH}}\left(t\right)|\Psi_{\beta }^{\mathrm{MCH}}\left(t+\Delta t\right)〉$$

The local diabatization algorithm is very stable in the case of trivial crossings (where NACs are extremely narrow and large; Plasser et al., 2012), and the fact that no NAC vectors need to be computed is attractive as it makes more quantum chemical methods amenable to SH. The local diabatization algorithm was actually the original inspiration for the three‐step propagator in SHARC (Mai, Marquetand, & González, 2015) and is the de facto standard way to propagate the electronic wave function in SHARC. To compute the required overlaps efficiently, SHARC2.0 comes with the wfoverlap program (Plasser et al., 2016), which makes extensive use of recurring intermediates, very efficient wave function truncation, and parallelization. This procedure was shown (Plasser et al., 2016) to be several orders of magnitude faster than a widely used previous implementation of such overlaps (Pittner, Lischka, & Barbatti, 2009).

The original fewest‐switches SH formula proposed by Tully (1990) involves the right‐hand side of the equation of motion (6). Within SHARC, one needs instead the right‐hand side of Equation (9), which contains the problematic derivative **U** ^†^ *∂* **U**/*∂t*. As the computation of this derivative should be avoided, another equation that adheres to the fewest‐switches principle is needed. In SHARC, the following equation is used by default, which again is inspired by the local diabatization method (Granucci et al., 2001):

(12) $$h_{\beta \to \alpha }=\left(1-\frac{{\left|c_{\beta }\left(t+\Delta t\right)\right|}^{2}}{{\left|c_{\beta }\left(t\right)\right|}^{2}}\right)\frac{ℜ\left[c_{\alpha }\left(t+\Delta t\right)P_{\alpha \beta }^{*}c_{\beta }^{*}\left(t\right)\right]}{{\left|c_{\beta }\left(t\right)\right|}^{2}-ℜ\left[c_{\beta }\left(t+\Delta t\right)P_{\beta \beta }^{*}c_{\beta }^{*}\left(t\right)\right]}$$

Alternatively, in SHARC2.0 hopping probabilities can also be computed with the “global flux SH” formula (Lisinetskaya & Mitrić, 2011; L. Wang, Trivedi, & Prezhdo, 2014), which might be more advantageous if “super‐exchange” mechanisms are present (i.e., if two states are only coupled via a classically forbidden third state).

In order to perform the dynamics simulations on the PESs of the diagonal states, it is necessary to compute the gradients corresponding to these states, based on the knowledge of the gradients of the MCH states and the transformation matrix. Similar to the gradient used in Ehrenfest dynamics (Doltsinis, 2006), the gradients of the diagonal states can be written as

(13) $$g_{\alpha }^{\mathrm{diag}}=\sum_{\mu }{\left|U_{\mu \alpha }\right|}^{2}g_{\mu }^{\mathrm{MCH}}+\sum_{\mu \neq \nu }U_{\mu \alpha }^{*}U_{\nu \alpha }\left[H_{\mu \mu }^{\mathrm{MCH}}-H_{\nu \nu }^{\mathrm{MCH}}\right]K_{\mu \nu }^{\mathrm{MCH}}$$

It can be seen that the full transformed gradient is a linear combination of several MCH gradients (first term), plus a contribution of the energy‐difference‐scaled NAC vectors (second term). The second term will significantly contribute if the NAC vector $K_{\mu \nu }^{\mathrm{MCH}}$ is large, which will usually occur close to conical intersections. However, the second term is negligible if all factors $U_{\mu \alpha }^{*}$ *U*_*να*_(*μ* ≠ *ν*) vanish, which will happen if in each column and row of **U** only one value is nonzero—this will happen if SOCs are small and no significant state mixing occurs. If the NAC vectors are not available from quantum chemistry or too expensive to compute, then it is often a good approximation to neglect the second term in the gradient, especially in systems without large SOCs (hundreds of cm^−1^). The quality of this approximation can be checked by monitoring the total energy conservation in the trajectories. Beyond the terms in Equation (13), it might be necessary to also include Hellmann–Feynman terms $〈\Psi_{\alpha }|\partial {\hat{H}}^{\text{additional}}/\partial R|\Psi_{\beta }〉$ in the gradient transformation—for example, if very heavy atoms dissociate from a molecule or if strong laser fields are present—although they tend to be difficult to obtain with electronic structure codes.

Another very important aspect which needs to be considered in SHARC simulations is the tracking of the absolute phase of all parts of the electronic wave function. This is important for two mathematical objects—one is the electronic basis functions computed in the quantum chemistry calculations, and the other is the transformation matrix **U**.

Regarding the basis functions, it is known that in general, wave functions computed with any quantum chemistry program have a (usually) random sign. This does not affect energies or gradients, but any off‐diagonal element $〈\Psi_{1}|\hat{O}|\Psi_{2}〉$ will change sign if one of the two state wave functions changes sign from one time step to the next. Within SHARC, these random sign changes are efficiently tracked through the computation of the state overlap matrix **S** (Equation (11)) and automatically removed. This always works as long as all matrix elements from the quantum chemistry software are based on the same wave functions and therefore any sign changes are consistent in all matrix quantities. The current interfaces in SHARC2.0 all provide this consistency, but one should nonetheless check the trajectories for random sign fluctuations in the involved quantities.

Regarding the transformation matrix **U**, each column of this matrix can be multiplied by a complex phase factor e^i*θ*^ and still be an eigenvector of **H** ^MCH^. Moreover, degenerate eigenvectors can freely mix, adding an arbitrary mixing angle to the set of undetermined parameters. This means that each numerical diagonalization during the dynamics could yield different, random phase factors which only depend on the implementation details of the diagonalization routine. These random phase factors would make **U** nonsmooth—a fact which is very detrimental to the electronic propagation and any subsequent analysis of the coefficients. Fortunately, in the above‐described three‐step propagator, the random phase factors cancel out during the propagation, so that tracking of the phase factors is not a big issue. Still, in our experience (Mai, Marquetand, & González, 2015), and as was pointed out by Pederzoli and Pittner (2017), it is preferable to perform phase tracking. The reason is that uncontrolled phase factors with the three‐step propagator can lead to random population transfer among the components of a multiplet, possibly leading to unnecessary random hops between the components (Pederzoli, & Pittner, 2017). The original phase tracking algorithm in SHARC (Mai, Marquetand, & González, 2015) was based on the overlap matrix between the **U** matrices of two subsequent time steps (**U** ^†^(*t*)**U** ^untracked^(*t* + Δ*t*)) and computes the phase‐tracked **U** matrix at *t* + Δ*t* as:

(14) $$U^{\text{tracked}}\left(t+\Delta t\right)=U^{\text{untracked}}\left(t+\Delta t\right)\hat{O}\hat{C}{\left[U^{†}\left(t\right)U^{\text{untracked}}\left(t+\Delta t\right)\right]}^{†}$$

where $\hat{O}$ Löwdin orthonormalizes the matrix it acts on. $\hat{C}$ makes the matrix in square brackets commute with **H** ^diag^(*t* + Δ*t*), which is achieved by setting all matrix elements to zero if they correspond to nondegenerate eigenstates in **H** ^diag^(*t* + Δ*t*) (Mai, Marquetand, & González, 2015). This algorithm was claimed to fail in some cases where multiple states cross simultaneously between two time steps (Pederzoli & Pittner, 2017) because the algorithm would assign the phases to the wrong states. Despite that such a situation is not likely to occur often in molecular systems, in SHARC2.0 (Mai, Richter, Heindl, et al., 2018), the algorithm has been improved by using the state overlap matrix **S** (Equation (11)) to locally diabatize and thus transform away any state crossings before applying the algorithm. This is possible because the state overlap matrix is real and thus does not affect the complex phases which need to be tracked; with this change, the algorithm can be stated as

(15) $$U^{\text{tracked}}\left(t+\Delta t\right)=U^{\text{untracked}}\left(t+\Delta t\right)\hat{O}\hat{C}{\left[U^{†}\left(t\right)S(t,t+\Delta t)U^{\text{untracked}}\left(t+\Delta t\right)\right]}^{†}$$

This approach fully corrects the algorithm for the (already rare) cases where the old one failed. In the case that **S** is not computed, SHARC2.0 falls back to the old algorithm. Yet, all current interfaces available within SHARC2.0 allow to compute the overlap matrix **S**.

**Figure 2:**
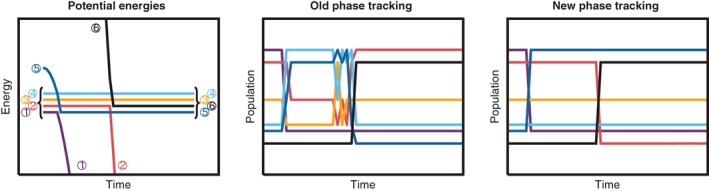
Energies and populations of a dynamics simulation with a model system showing the necessity of doing phase corrections to the matrix **U**. The left panel shows the energies of the six involved states, ordered by energy as is done in Surface Hopping including ARbitrary Couplings (SHARC). Note that in the panel, the energy differences between the multiplet components are arbitrarily increased for clarity; in the computations, they were degenerate. The middle panel shows the evolution of the populations when phase tracking is performed with the old tracking algorithm in SHARC. The right panel shows the populations with the new tracking algorithm of SHARC2.0, delivering the expected result that at the crossing points complete population transfer occurs between the crossing state pairs, whereas the other states are not affected

The effect of the new tracking algorithm is exemplified in Figure 2, where we repeated the computations of Pederzoli and Pittner (2017). The computation is based on a model of two coupled harmonic oscillators which cross with an uncoupled set of four degenerate states. Pederzoli and Pittner (2017) showed that in this model, the old tracking algorithm of SHARC failed, as shown in the middle panel. Conversely, the new tracking algorithm (right panel) produces the expected result of full population transfer when the harmonic oscillator states cross with the multiplet. We note, however, that the shown model (fully uncoupled multiplet with nonzero population) is very different from typical SHARC applications, where uncoupled states are typically omitted for efficiency reasons, and where population in uncoupled states is quickly collapsed by the decoherence correction schemes.

### 3.4 Alternative approaches

The general field of SH has been flourishing for more than two decades. As a consequence, there exist a large number of codes for this aim, such as Newton‐X (Barbatti et al., 2014), JADE (Du & Lan, 2015), PYXAID (Akimov & Prezhdo, 2013), or ANT (Zheng et al., 2017). Furthermore, many quantum chemistry suites (e.g., MOLCAS: Aquilante et al., 2015; TURBOMOLE: Furche et al., 2014; or CPMD, 2017) and molecular dynamics suites (e.g., ChemShell: Metz, Kästner, Sokol, Keal, & Sherwood, 2014) incorporate a basic SH module. However, the field of SH methods which can treat additional couplings in the Hamiltonian is much smaller.

In the field of SH dynamics targeted at the simulation of ISC processes, one can distinguish between three general approaches. The oldest one combines regular Tully SH with ISC probabilities computed only at the relevant crossing points, using transition probabilities from Landau‐Zener (Zener, 1932) or Zhu‐Nakamura (Ishida, Nanbu, & Nakamura, 2017) theory to estimate the singlet‐to‐triplet transition probability. Such procedure has been used for different kinds of collision reactions involving ISC (Abrahamsson, Andersson, Marković, & Nyman, 2009; Amatatsu, Morokuma, & Yabushita, 1991; Hu, Lendvay, Maiti, & Schatz, 2008; Rajak & Maiti, 2014; Tachikawa, Ohnishi, Hamabayashi, & Yoshida, 1997; Yamashita & Morokuma, 1990).

In the second approach, SOCs are properly incorporated into the electronic equation of motion. This can lead to population flux from one multiplicity to another, possibly inducing surface hops. However, in this approach, the PESs are not modified by the SOCs, which means that the dynamics is carried out on the surfaces corresponding to MCH states. Some authors refer to this approach as SH on “adiabatic/spin‐diabatic” surfaces (Granucci et al., 2012). The fact that the PES are not affected by SOCs has the advantage that the SH algorithm requires minimal changes—the only difference to regular SH is that in Equation (6) the Hamiltonian matrix elements *H*_*αβ*_ do not form a diagonal matrix. This approach has been implemented in several SH codes published in the last years. For example, Franco de Carvalho and Tavernelli (2015) reported SH simulations with ISC for SO_2_ using CPMD (2017) and LR‐TDDFT electronic structure. As one variant of the “spin‐diabatic” approach, they treat each multiplet as only one effective state, that is, they do not consider separate coefficients *c*_*α*_(*t*) (Equation (1)) for all the multiplet components. The employed “effective” SOC matrix elements are obtained as the sum of all SOC elements between the involved multiplet components. According to Granucci et al. (2012) and in our experience, this effective SOC approach should be treated with caution, as the electronic propagation is significantly different from a propagation including all components. This might be the reason that in SO_2,_ they find significant ISC to all three relevant triplet states (*T*_1_ to *T*_3_), whereas other works show that only one of these triplets is populated due to symmetry (Lévêque, Taïeb, & Köppel, 2014; Mai, Marquetand, & González, 2014; Xie, Hu, Zhou, Xie, & Guo, 2013). The same general approach—SH in the “spin‐diabatic” basis with merged multiplet components—is also implemented in the generalized trajectory SH method of Cui and Thiel (2014). Other related work using a “spin‐diabatic” basis was reported by Habenicht and Prezhdo (2012) to study ISC in carbon nanotubes. Moreover, some earlier works on collision reactions performed SH including ISC on fully diabatic potentials (Fu, Shepler, & Bowman, 2011; Han & Zheng, 2011). We also note that in a number of more recent methods—like generalized ab initio multiple spawning (Curchod, Rauer, Marquetand, González, & Martínez, 2016; Fedorov, Pruitt, Keipert, Gordon, and& Varganov, 2016) or direct‐dynamics vibrational multiconfigurational Gaussian (Richings et al., 2015)—the “spin‐diabatic” basis is also used. However, the latter methods include more quantum mechanical effects and hence might not show the same dependence on representation as SH does.

As discussed above, the optimal basis for SH is the diagonal basis, where the SOCs directly affect the shape of the PES. Hence, the third general approach for SH including ISC is to use the diagonal basis, which some authors refer to as “spin‐adiabatic” basis (Granucci et al., 2012). Besides SHARC and some early applications to scattering reactions (Maiti, Schatz, & Lendvay, 2004), some of the earliest followers of this approach were Persico and coworkers (Granucci et al., 2012). They comprehensively showed that the spin‐diabatic approach is incorrect because the effective SOC elements ignore the direction of the SOC vectors (i.e., containing the SOCs between all components of the involved multiplets), which become important if more than one singlet and one triplet are considered. Alternatively, if in the spin‐diabatic approach, multiplet components are treated explicitly, then the approach does not guarantee rotational invariance of the results. Despite its clear superiority, the spin‐adiabatic/diagonal approach is not yet widely spread. SHARC employs the diagonal basis since its birth in 2011, but SH in the diagonal basis has been implemented in the otherwise long‐established Newton‐X only in 2017 by Pederzoli and Pittner (2017). These authors have also introduced two new propagators in an effort to solve the problems with arbitrary phases in the matrix **U**. However, for these propagators, it is noted that the employed modified **U** matrices do not diagonalize the Hamiltonian—a fact which could be problematic if they couple hopping probabilities in the nondiagonalizing basis with nuclear gradients in the diagonal basis, because in this way electronic wave function and nuclear potentials could become inconsistent. On the contrary, if they employ gradients in the nondiagonalizing basis their algorithm loses the above‐mentioned benefits of the diagonal basis. Hence, the locally diabatic phase tracking of diagonal states in SHARC2.0 (Equation (15) and Figure 2) should be a safer solution to the arbitrary phase problem.

On a side note, we recall that SHARC can also be used to study molecules under the influence of electric fields. Analogously to the case of ISC dynamics, there are three general approaches which can be used for SH in this case. In the first, the dipole couplings can be simply added to the electronic Hamiltonian in the equation of motion (6), but with the nuclear dynamics evolving on field‐free potentials. This approach—the counterpart of the “spin‐diabatic” approach for ISC—is conceptually simple (Jones, Acocella, & Zerbetto, 2008) and it has been popularized by the field‐induced SH method of Mitrić et al. (2009) and Mitrić, Petersen, Wohlgemuth, Werner, and Bonačić‐Koutecký (2011), followed by a number of related implementations (Bajo et al., 2014; Tavernelli, Curchod, & Rothlisberger, 2010). The same approach also forms the basis of the external‐field ab initio multiple spawning method (Mignolet, Curchod, & Martínez, 2016).

A second approach is to include the effect of dipole couplings on the PES. This idea was already proposed in the 1990s (Dietrich, Ivanov, Ilkov, & Corkum, 1996; Kelkensberg, Sansone, Ivanov, & Vrakking, 2011; Thachuk et al., 1996, 1998), is in principle equivalent to the “spin‐adiabatic” approach, and is the one implemented in SHARC (Richter et al., 2011) and used in the early laser field applications (Marquetand et al., 2011). Other authors refer to this approach as SH on field‐dressed states (Thachuk et al., 1996, 1998).

The third approach for SH including laser fields is to perform SH on PES obtained after diagonalizing the electronic Hamiltonian in the Floquet picture (Bajo et al., 2012; Fiedlschuster et al., 2017; Fiedlschuster, Handt, & Schmidt, 2016). The advantage is that in this picture, the PES do not change as rapidly as in the “field‐dressed” SH approach, where they change depending on field frequency. On the contrary, in the Floquet picture the potentials change only depending on the envelope function of the pulse. Recently, it was shown that Floquet‐based SH delivers much better results than field‐free or field‐dressed SH when compared to exact dynamics (Fiedlschuster et al., 2017, 2016). Although the Floquet picture was used in one early application of the SHARC approach (Bajo et al., 2012), it is not currently implemented in SHARC2.0. However, Floquet‐based SH can only be applied to laser fields where the Floquet treatment is appropriate. Rigorously, this is only the case if the laser field is strictly time periodic, but the approach is still approximately correct for fields with constant central frequency and slowly varying envelope.

## 4 THE SHARC2.0 DYNAMICS SUITE

### 4.1 Organization of the SHARC2.0 suite

The SHARC2.0 program suite (Mai, Richter, Heindl, et al., 2018) provides an implementation of the SHARC approach together with a large set of auxiliary programs for various tasks such as setup, analysis, or quantum chemistry interfacing. The core program of SHARC2.0—the dynamics driver sharc.x—is written in Fortran 90, whereas most auxiliary programs are written in Python 2. The wave function overlap program WFOVERLAP (Plasser et al., 2016), which is essential for most ab initio dynamics simulations using SHARC2.0, is also written in Fortran 90.

**Figure 3:**
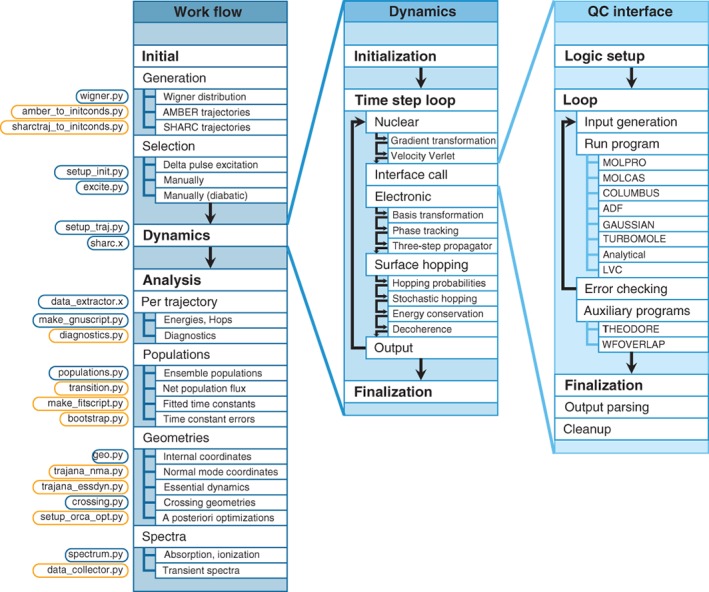
Work flow of SHARC2.0 dynamics simulations. The left column shows the work flow on the ensemble level, including the preparation (initial conditions), dynamics, and analysis steps, each with applicable options. The labels on the left give the names of the SHARC2.0 subprogram, which performs the respective task. Subprograms which are new with respect to the original implementation of SHARC are marked in orange. The middle column shows the work flow inside the dynamics driver, on a trajectory level. The right column shows, on the time step level, the work flow of the quantum chemistry (QC) interface

The different parts of SHARC2.0 are presented in Figure 3 together with the general work flow during a full dynamics simulation project. The three columns in the figure show the work flow on three levels: (a) the ensemble level, where multiple trajectories are prepared, run, and analyzed; (b) the trajectory level, where nuclei and electrons are propagated from time step to time step; and (c) the time step level, where the quantum chemistry interfaces drive the electronic structure calculations. On the left of the figure, the different programs in the SHARC2.0 suite are given, next to the task they perform.

### 4.2 Generation of initial conditions

The generation of initial conditions in SHARC2.0 consists of two general steps. In the first one, a large number of initial geometries and corresponding initial velocities are generated. For rather small, rigid molecules in the gas phase, the preferred approach is to sample geometries and velocities from a Wigner distribution of the ground state harmonic oscillator (Barbatti & Sen, 2016; Dahl & Springborg, 1988; Schinke, 1995). The effect of high temperature can be included by sampling from a Boltzmann‐weighted combination of different vibrational states of the harmonic oscillator. This approach usually produces distributions of coordinates and energy which are close to the actual quantum distributions (Barbatti & Sen, 2016).

Unfortunately, Wigner sampling cannot be applied to large and flexible systems with many degrees of freedom, such as those containing long alkane chains or flexible groups, biopolymers, or simply molecules in solution. The reason is that these systems possess a large number of local minima in the ground state PES as well as many anharmonic, nonlinear vibrational modes such as torsions or solvent diffusion, making the linear harmonic oscillator approximation invalid for these systems. Initial conditions for such systems can be prepared by running sufficiently long molecular dynamics simulations in the ground state and extracting snapshots from the trajectory (Garavelli et al., 2001). Within the SHARC2.0 suite, currently one can convert the results of AMBER (Case et al., 2017) simulations to the native SHARC format to create such initial conditions. Alternatively, initial conditions can be sampled from previous SHARC trajectories. The latter is not only useful to obtain initial conditions, but can also be used to restart excited‐state trajectories with modified settings, for example, reducing the number of states after initial relaxation, switching level of theory, or following ground state dynamics after relaxation with single‐reference methods.

The second step of preparing initial conditions in SHARC2.0 is to assign for each initial geometry, the corresponding initial electronic state. This state can be specified manually by the user, using either the MCH or the diagonal basis, or in a quasi‐diabatic basis obtained through overlap computations between the initial geometry and a reference geometry with known electronic states. However, the more common procedure is to perform a single point calculation for each initial geometry and select the initial state stochastically (Barbatti et al., 2007), based on the obtained excitation energies, oscillator strengths, and some assumptions about the excitation process, for example, coming from experimental setups. Within SHARC, this stochastic selection process can either be carried out in the diagonal or the MCH basis, although for the employed delta pulse approximation only the MCH basis is rigorously correct.

### 4.3 Dynamics driver

After preparation of the initial conditions, the SHARC trajectories are ready to be executed. The SHARC2.0 dynamics driver offers several popular algorithms for the coupled propagation of nuclei and electrons. The nuclei are generally propagated with the velocity‐Verlet algorithm (Verlet, 1967). It is possible to propagate the nuclei on either MCH or diagonal PESs, although the latter is generally recommended. In that case, the nuclear gradients are computed by a transformation of the MCH gradients, as given in Equation (13). The contribution of the NACs to the gradient can optionally be neglected, if NACs are not available or to speed up the calculations.

The electronic wave function is propagated using the three‐step propagator approach (Equation (10)). The MCH propagator **P** ^MCH^(*t* + Δ*t*, *t*) is computed as a product of matrix exponentials, using shorter time steps than in the nuclear propagation and linear interpolation of all quantities (Mai, Marquetand, & González, 2015). One can either employ the standard approach, which includes the NAC contribution **v** ·**K** ^MCH^, or the local diabatization approach (Granucci et al., 2001), which works with the wave function overlap matrix **S** (Equation (11); Plasser et al., 2016). Wave function and transformation matrix phases are always automatically tracked, as explained above.

The dynamics driver also carries out all steps related to the SH. Hopping probabilities can either be computed with Equation ((12); Mai, Marquetand, & González, 2015) or with global flux SH (L. Wang et al., 2014). Two decoherence correction schemes are available: the energy‐based correction suggested by Granucci and Persico (2007) and the augmented SH algorithm put forward by Jain et al. (2016), which is based on propagating auxiliary trajectories for the nonactive states. Kinetic energy adjustment after a hop can either be omitted, or done parallel to the full velocity vector or the relevant NAC vector. In either case, frustrated hops can be treated with or without reflection. For QM/MM calculations, it is also possible to consider the kinetic energy of only a subset of atoms for decoherence correction and kinetic energy adjustments.

### 4.4 Quantum chemistry interfaces

The dynamics driver is completely separated from the quantum chemistry programs through a file‐based interface, making the driver unaware of which electronic structure method is employed. SHARC2.0 is currently interfaced with six quantum chemistry programs—MOLPRO (Werner, Knowles, Knizia, Manby, & Schütz, 2012; Werner et al., 2012), MOLCAS (Aquilante et al., 2015), COLUMBUS (Lischka et al., 2011, 2012), ADF (Baerends et al., 2017), GAUSSIAN (Frisch et al., 2016), and TURBOMOLE (Furche et al., 2014; TURBOMOLE, 2015). These interfaces allow carrying out SHARC simulations with the state‐averaged complete active space self‐consistent field (SA‐CASSCF; Roos, Taylor, & Siegbahn, 1980), multistate CAS second‐order perturbation theory (MS‐CASPT2; Andersson, Malmqvist, Roos, Sadlej, & Wolinski, 1990), multi‐reference configuration interaction including singles and doubles (MR‐CISD; Shepard, Lischka, Szalay, Kovar, & Ernzerhof, 1992), time‐dependent density functional theory (TD‐DFT; Ferré, Filatov, & Huix‐Rotllant, 2016), algebraic diagrammatic construction to second order (ADC(2); Dreuw & Wormit, 2015; Trofimov & Schirmer, 1995), and approximate coupled cluster with doubles (CC2; Hättig, 2005). Two additional interfaces provide engines to drive dynamics on analytical model potentials, including general linear‐vibronic coupling (LVC) models (Köppel, Domcke, & Cederbaum, 1984; Plasser, Goméz, Mai, & González, 2018). Because the dynamics driver and the quantum chemistry programs are independent of each other, it is very easy to develop new interfaces for other electronic structure methods or codes. The capabilities of the SHARC2.0 interfaces are summarized in Table 1.

**Table 1:** Overview over the capabilities of the SHARC2.0 interfaces. For each method and program, the table shows which multiplicities (*S* ^2^), which quantities, and whether QM/MM partition are available

Method	Program	*S* ^2^	SOC	TDM^a^	Grad.	NAC	OVL^b^	ION	Theo.^c^	QM/MM
SA‐CASSCF	MOLPRO	Any	✓	✓	✓	✓	✓	✓		
	MOLCAS	Any	✓	✓	✓		✓	✓		✓
	COLUMBUS	Any	✓^e^	✓	✓	✓^e^	✓	✓		
MS‐CASPT2	MOLCAS	Any	✓	✓	✓^d^		✓	✓		
MR‐CISD	COLUMBUS	Any	✓^e^	✓	✓	✓^e^	✓	✓		
TD‐DFT	ADF	Any	✓^f^	✓^g^	✓		✓	✓	✓	✓
	GAUSSIAN	Any		✓^g^	✓		✓	✓	✓	
ADC(2)	TURBOMOLE	S, T	✓^f^	✓	✓		✓		✓	
CC2	TURBOMOLE	S, T		✓^g^	✓		✓		✓	
Analytical	—	Any	✓	✓	✓		✓			
LVC	—	Any	✓	✓	✓	✓	✓			

a TDM: transition dipole moments.

b OVL: wave function overlaps.

c Theo.: TheoDORE.

d Numerical gradients.

e Either NAC or SOC, but not both at the same time.

f SOCs only between singlets and triplets.

g TDMs only between *S*_0_ and excited singlets.

In case of computationally demanding systems, some interfaces take advantage of the parallel computing capabilities of the quantum chemistry programs (ADF, GAUSSIAN, and TURBOMOLE). Additionally, most interfaces can automatically schedule and distribute independent parts of the calculations to different CPUs, for example, wave function computations for several multiplicities, gradient computations for several states, or displacements for numerical gradients, in order to save wall clock time.

### 4.5 Trajectory and ensemble analysis

The analysis of the simulation results can be performed in two general ways, either by manually analyzing individual trajectories or by statistical analysis of the ensemble. Although the latter is arguably more important, both approaches have their value. Individual analysis of trajectories is required to verify that all considered trajectories are physically sound and is a good basis for hypothesis building. Ensemble analysis can then be used to test these hypotheses and will provide most chemically interesting conclusions.

The quantities that can be analyzed in SHARC are divided into two groups (Persico & Granucci, 2014; Tully, 1990). The first group are physical observables. The most prominent examples are quantum yields, which can be defined either through the population of specific electronic states—ground state, long‐lived triplet states, ionic states—or through nuclear coordinates, like in rearrangement reactions. The time evolution of certain quantum yields can be compared to experimentally measured lifetimes. For dissociation and scattering reactions, it is also possible to obtain velocity or kinetic energy distributions from the simulations. Other observables that can be obtained from the trajectories are different kinds of transient signals, such as transient absorption spectra (Berera, Grondelle, & Kennis, 2009), time‐resolved photoelectron spectra (Stolow, Bragg, & Neumark, 2004), time‐resolved infrared spectra (Nibbering, Fidder, & Pines, 2005), or time‐dependent nuclear distribution functions (Bressler & Chergui, 2010; Sciaini & Miller, 2011). The second group of quantities that can be analyzed are descriptors (Persico & Granucci, 2014; Tully, 1990). These are not physical observables but are very useful for formulating reaction mechanisms, generalizing to classes of molecules, or comparing to other computational simulations. Examples are the character of electronic wave functions or internal nuclear coordinates.

The SHARC2.0 suite contains a number of tools which aid in the analysis of individual trajectories, in the detection of problems occurring in the simulations, and in the ensemble analysis. Electronic populations can be computed with a number of protocols, for example, by (a) summing up the diagonal quantum amplitudes |$c_{\alpha }^{\mathrm{diag}}$(*t*)|^2^ over all trajectories or by (b) counting the numbers of trajectories in each diagonal state. By transforming the quantum amplitudes into the MCH representation, that is, ${\left|c_{i}^{\mathrm{MCH}}\left(t\right)\right|}^{2}={\left|\sum_{i}U_{\mathrm{i\alpha}}\left(t\right)c_{\alpha }^{\mathrm{diag}}\left(t\right)\right|}^{2}$, the sum of quantum amplitudes can also be computed for the spin‐free MCH states (c), which is usually very helpful in interpreting the populations. Using this transformation to obtain the number of trajectories in each MCH state (d) can only be done in an approximate way. One can also (e) compute quasi‐diabatic populations (Mai, Marquetand, & González, 2014), or (f) use histogram binning (Mai, Marquetand, Richter, González‐Vázquez, & González, 2013), for example, to compute the number of trajectories whose oscillator strength is above 0.1. The population plots, together with monitoring population flux between the states, allow proposing kinetic models for the observed photoreactions. Additional tools allow fitting these kinetic models to the population data and to compute errors (Nangia et al., 2004) for the obtained kinetic parameters. These computed errors can be used to verify that a sufficient number of trajectories were computed for the employed population protocol and kinetic model. The evolution of the electronic wave function can also be monitored on‐the‐fly through charge transfer numbers computed with the TheoDORE package (Plasser, 2017; Plasser, Wormit, & Dreuw, 2014). The nuclear evolution can be analyzed through internal coordinates (bond lengths, angles and dihedrals), through normal mode coordinates (Kurtz, Hofmann, & de Vivie‐Riedle, 2001; Plasser, 2009), or through essential dynamics analysis (Amadei, Linssen, & Berendsen, 1993). Furthermore, it is possible to extract automatically the geometries where hops between specific state pairs occur. Naturally, as the trajectory analysis is the most important and most specific step, users might need to carry out specific analysis procedures depending on the application, which might not be catered for in the general tools of SHARC2.0. However, all results in SHARC2.0 are stored in human‐readable text files, allowing easy access to all raw data.

A posteriori investigations of the PES for the interpretation of mechanisms are assisted by tools that allow optimization of minima and crossing points for all excited states encountered during the SHARC simulations. This is facilitated by an interface between the ORCA (Neese, 2012) external optimizer and the SHARC2.0 suite, which delivers necessary energies and gradients (Bearpark, Robb, & Schlegel, 1994; Levine, Coe, & Martínez, 2008) to ORCA. This interface allows optimizations that often are not possible within other quantum chemistry programs. In this way it is, for example, possible to optimize crossing points using gradients from ADF or TURBOMOLE, or from GAUSSIAN with TD‐DFT.

## 5 SHARC APPLICATION EXAMPLES

In the first publication of the SHARC method (Richter et al., 2011), both the influence of field–dipole couplings and SOCs were tested in an analytical model of the IBr molecule. Further tests included strong, off‐resonant laser interactions via the so‐called nonresonant dynamic Stark effect in the same model (Marquetand et al., 2011). Ideally, such interactions are treated in the Floquet picture, where the PESs of relevant states are replicated as many times as photons are considered to interact with the molecule in order to include multiphoton processes. This approach was exemplified in an analytical model of the Na_2_ system (Bajo et al., 2012).

The first application of the SHARC method in an on‐the‐fly framework was devoted to the investigation of the excited‐state dynamics of the nucleobase keto‐amino cytosine in gas phase (Mai et al., 2013; Richter, Marquetand, González‐Vázquez, Sola, & González, 2012b). It was found that ISC to the triplet states can take place on a femtosecond timescale, competing with the well‐known ultrafast IC pathways to the electronic ground state (Barbatti, Borin, & Ullrich, 2015; Crespo‐Hernández, Cohen, Hare, & Kohler, 2004; Middleton et al., 2009). Despite rather weak SOCs (typicall about 20–40 cm^−1^)—as expected for second row element atoms—the very small energetic separation between the singlet and triplet states led to efficient ISC on an ultrafast time scale. In Mai et al. (2013), the enol‐amino tautomer of cytosine was also investigated. In comparison to the keto‐amino tautomer, the enol‐amino tautomer shows only negligible ISC. Also other relaxation processes, such as IC, happen on different time scales in the two tautomeric forms and a rather complex picture of the excited‐state dynamics is obtained. This intricate dynamics can lead to enormous complications in attributing experimentally found time scales to the calculated molecular processes, as detailed by Ruckenbauer, Mai, Marquetand, and González (2016).

Related works using SHARC for nucleobases were focused on uracil (Richter & Fingerhut, 2016; Richter, Mai, Marquetand, & González, 2014) and thymine (Mai, Richter, Marquetand, & González, 2015a, 2017), where also a low but non‐negligible population of triplet states was observed in gas phase. Generally speaking, these works show that in the isolated pyrimidine nucleobases the triplet states can potentially be relevant to understand its excited‐state relaxation dynamics, and that they should not be neglected a priori.

Driven by our interest in nucleobases and their reaction to ultraviolet irradiation, the role of triplet states on the thymine dimer formation—one of the most abundant DNA photolesions—was investigated using SHARC. Interestingly, the nonadiabatic simulations performed showed (Rauer, Nogueira, Marquetand, & González, 2016) that triplet states remain unpopulated along the reaction pathway on an ultrafast timescale. In contrast to the direct formation of the thymine dimers, the role of triplet states is well documented when a photosensitizer, which prepares the system containing two thymines directly in a triplet state, is employed. Using SHARC in conjunction with other methods, it could be elucidated that the thymine dimer formation in the triplet manifold is a stepwise reaction mechanism, where a long‐lived triplet biradical intermediate is traversed before a bifurcation on the ground‐state PES leads to the cyclobutane photoproduct with low yield (Rauer, Nogueira, Marquetand, & González, 2018).

**Figure 4:**
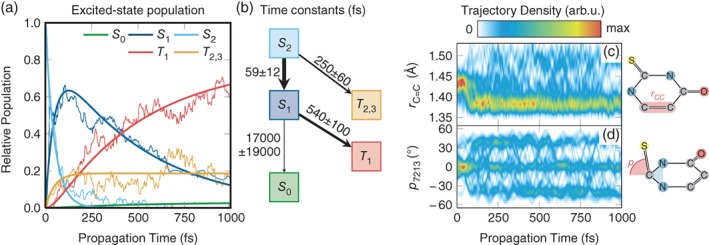
Overview over results obtained from SHARC simulations for 2‐thiouracil using the MS‐CASPT2(12,9)/cc‐pVDZ method (Mai, Marquetand, & González, 2016). In (a), the time‐dependent populations (thin lines) and kinetic model fits (thick lines). In (b), the assumed kinetic model with the obtained fit parameters and errors. In (c) and (d), the temporal evolution of two key geometric parameters (C=C bond length and thiocarbonyl pyramidalization angle). (Reprinted with permission from Mai, Marquetand, and González (2016). Copyright 2016 ACS, published under CC‐BY license)

SHARC was also applied to a large number of modified nucleobases, whose chemical formulae differ only slightly from the canonical nucleobases but whose excited‐state dynamics can be dramatically different (Mai et al., 2016; Pollum, Martínez‐Fernández, & Crespo‐Hernández, 2015). In this regard, thio‐substituted nucleobases (thiobases)—bearing a sulfur atom instead of an oxygen atom—are probably among the most interesting systems. Unlike their canonical counterparts, thiobases show exceptionally high‐ISC yields, usually in the range of 90–100%. With SHARC, the excited‐state dynamics of 2‐thiouracil (Mai, Marquetand, & González, 2016; Mai et al., 2017) and 2‐thiocytosine (Mai, Pollum, et al., 2016) was investigated. Figure 4, which is adapted from Mai, Marquetand, and González (2016), shows an example of the time evolution of the singlet and triplet states in 2‐thiouracil. The figure also presents the kinetic model assumed for the shown fit, including the obtained time constants for the IC and ISC processes. On the right panel, the figure also depicts the temporal evolution of two important internal coordinates, which are very helpful in analyzing the dynamics in the two triplet minima of 2‐thiouracil (Koyama, Milner, & Orr‐Ewing, 2017; Mai, Marquetand, & González, 2016; Sanchez‐Rodriguez et al., 2017). Both mentioned thiobases showed ISC in the time range of a few 100 fs, consistent with experimental results on these two and several other thiobases (Koyama et al., 2017; Mai, Pollum, et al., 2016; Martínez‐Fernández, Corral, Granucci, & Persico, 2014; Pollum, Jockusch, & Crespo‐Hernández, 2014, 2015; Pollum, Martínez‐Fernández, & Crespo‐Hernández, 2015; Pollum, Ortiz‐Rodríguez, Jockusch, & Crespo‐Hernández, 2016; Sanchez‐Rodriguez et al., 2017). Based on the SHARC results, a general explanation for this behavior of thiobases was put forward (Mai, Pollum, et al., 2016), stating that in thiobases the excited‐state minima are stabilized by thionation, whereas the *S*_1_/*S*_0_ conical intersections retain the same energies as in the canonical bases. As a consequence, there is a very large barrier for ground state relaxation, making ISC the only viable deactivation route in these molecules and explaining the exceptionally high‐ISC yields. Other nucleobase analogues—purine (Crespo‐Hernández et al., 2015), 6‐azacytosine (Borin, Mai, Marquetand, & González, 2017), or 5‐bromouracil (Peccati, Mai, & González, 2017)—were also investigated with SHARC, showing that purine and 6‐azacytosine do not exhibit ultrafast ISC.

SHARC has also been used to study the excited‐state relaxation of the SO_2_ molecule (Mai, Marquetand, & González, 2014)—a system that has raised a lot of attention in the last years (Franco de Carvalho & Tavernelli, 2015; Wilkinson et al., 2014; Xie et al., 2013)—and the results agree nicely with independently published (Lévêque et al., 2014) exact quantum dynamics simulations on potentials of slightly higher accuracy. In particular, out of the three low‐energy triplets of SO_2_, only one is significantly populated due to symmetry reasons, and this is nicely reproduced in the SHARC simulations (Mai, Marquetand, & González, 2014). Furthermore, the release of singlet oxygen from cyclohexadiene endoperoxide (Martínez‐Fernández, González‐Vázquez, González, & Corral, 2014) was investigated and it was found that among the two competing pathways—cycloreversion and O–O homolysis—the latter is the dominant one with remarkable ISC efficiency. The mechanism of other photosensitizers was also investigated with SHARC, for example, the prototypical photosensitizer benzophenone (Marazzi et al., 2016), which can be used to promote thymine dimerization. That dynamical study showed that two discussed ISC mechanisms, involving the two lowest triplet states, coexist in a kinetic equilibrium. In the thiophene molecule (Schnappinger et al., 2017), photoexcitation leads to both ring puckering and ring opening followed by an interplay of IC and ISC due to the near degeneracy of several states. Furthermore, SHARC was also used to shed light on the ultrafast ISC pathways of 2‐nitronaphtalene (Zobel, Nogueira, & González, 2018, rationalizing the high‐ISC efficiency by virtue of small electronic and nuclear alterations of the chromophore when going from the singlet to the triplet manifold.

**Figure 5:**
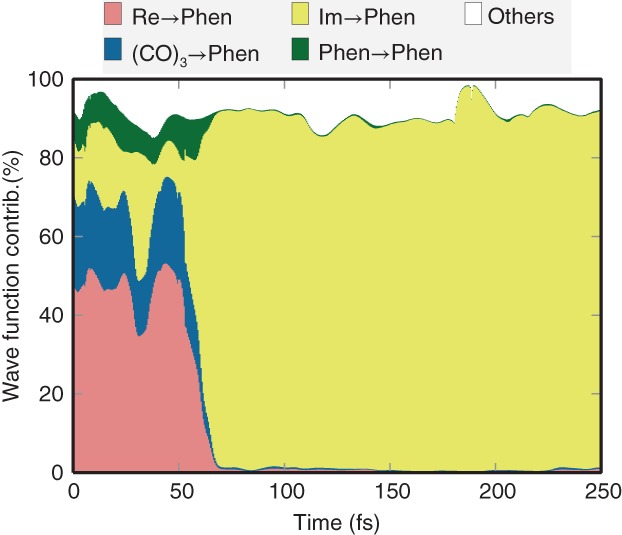
Time‐dependent wave function composition of an exemplary trajectory of [Re(CO)_3_(Im)(Phen)]^+^ in water. The different colors indicate the contributions of different charge transfer classes to the electronic wave function. Initially, the trajectory is predominantly in a Re(CO)_3_→ Phen (MLCT) state, but around 60 fs it converts to a very pure Im→Phen (LLCT) state

Quite recently, SHARC was interfaced to ADF (Baerends et al., 2017), which is one of the few density functional theory codes that can perform perturbational spin–orbit computations, and hence is ideally suited to study ISC phenomena. With SHARC and ADF, Atkins and González (2017) recently investigated the ultrafast dynamics of [Ru(bpy)_3_]^2+^, a prototypical transition metal complex widely utilized as a photosensitizer in photovoltaic and other photonic applications. These were the first trajectories using SOCs on‐the‐fly for a transition metal complex. They showed that the ultrafast ISC, taking place on a 25‐fs time scale, is not only due to the high density of states and the large SOCs (>400 cm^−1^), but requires nuclear relaxation involving Ru‐N bond vibrations, among other degrees of freedom (Atkins & González, 2017). In general, but particularly in transition metal complexes with their high density of states, it is extremely beneficial to follow the character of the electronic wave function on‐the‐fly (Mai et al., 2018). It is in this respect that the automatic characterization of charge transfer numbers using the TheoDORE code (Plasser, 2017; Plasser et al., 2014) can be extremely revealing. Figure 5 illustrates for one exemplary trajectory of [Re(CO)_3_(im)(phen)]^+^ (im = imidazole, phen = phenanthroline) in water the evolution of electronic wave function from predominantly Re(CO)_3_→ Phen (metal‐to‐ligand charge transfer) to Im→Phen (ligand‐to‐ligand charge transfer).

SHARC has a growing number of users, as documented by various publications from other research groups. Corrales et al. (2014) studied bond breaking times for alkyl iodides with alkyl chains of different lengths. They observed a linear relationship between the reduced mass of the chain and the bond breaking time, using a modified version of SHARC. A subset of the same authors employed the same approach for investigating the photodissociation of chloroiodomethane (Murillo‐Sánchez et al., 2017). Cao, Xie, and Yu (2016) ruled out the participation of previously proposed triplet intermediates in the N, O rearrangement reaction of oxazole and instead proposed singlet pathways. Cao (2018) also investigated the role of ring puckering and ring opening in the photorelaxation of thiazole and isothiazole using a modified SHARC‐MOLPRO interface. Banerjee, Halder, Ganguly, and Paul (2016) studied the electron‐catalyzed photofragmentation of 5‐phenyl‐2H‐tetrazole, in which upon photoexcitation, an electron is injected from one part of the molecule into another part, where bond dissociation takes place, and afterward the electron returns to its originating part of the molecule. Bellshaw et al. (2017) showed that the dynamics of the CS_2_ molecule is strongly affected by SOCs, as the dissociation barrier is much smaller in the triplet states than in the singlet ones. Siouri, Boldissar, Berenbeim, and de Vries (2017) used SHARC to identify ISC pathways in the photorelaxation of 6‐thioguanine tautomers. Pederzoli and Pittner (2017) investigated ISC processes in thiophene, as mentioned in the SHARC section above. Squibb et al. (2018) found out that, according to SHARC calculations based on CASSCF electronic structure properties, triplet states play a role in the photofragmentation of acetylacetone.

## 6 CONCLUSIONS

We have presented the SHARC approach, as it is implemented in the SHARC2.0 program package (Mai, Richter, Heindl, et al., 2018). The SHARC approach is an extension of the popular SH method, which allows simulating the full‐dimensional excited‐state dynamics of molecules including IC. With the SHARC approach, it is possible to incorporate arbitrary coupling terms in the electronic Hamiltonian, opening up the possibility to treat also other processes beyond IC, such as ISC or laser‐induced excitation.

The central idea of SHARC is that SH should be performed on the PESs of the eigenstates of the total electronic Hamiltonian, in contrast to many other SH approaches, where the eigenstates of the MCH are used. The eigenstates of the total electronic Hamiltonian are computed by diagonalization of the Hamiltonian matrix obtained from quantum chemistry. This diagonalization step makes it necessary to perform a number of basis transformations, which affect most of the working equations in SH. The working equations in SHARC are designed for optimal numerical accuracy and stability, which is one of the biggest achievements of the SHARC approach.

We have also provided a brief overview over the SHARC2.0 package, which has been released in the beginning of 2018. The core program of the new version of the SHARC package is the SHARC2.0 dynamics driver, which is currently interfaced to six quantum chemistry packages—MOLPRO (Werner, Knowles, Knizia, Manby, & Schütz, 2012), MOLCAS (Aquilante et al., 2015), COLUMBUS (Lischka et al., 2011), ADF (Baerends et al., 2017), GAUSSIAN (Frisch et al., 2016), and TURBOMOLE (Furche et al., 2014)—enabling dynamics simulations based on many popular electronic structure methods. The SHARC2.0 package also contains a large number of auxiliary programs, which automatize all steps in the preparation of the simulations and provide a wide array of analysis tools.

Finally, we have shown that the SHARC approach (in its previous implementation, Mai, Richter, et al., 2014) has been very successful in describing many excited‐state phenomena in a variety of molecular systems. Some highlights include the work on nucleobases and nucleobase analogous, the simulation of ISC in transition metal complexes such as [Ru(bpy)_3_]^2+^, and diverse works on small inorganic and organic chromophores.

As mentioned above, one of the most important ingredients for any SHARC simulation is an appropriate and efficient electronic structure method, which can facilitate accurate simulations over sufficiently long time scales and statistically large number of trajectories. Hence, a constant focus of the ongoing SHARC development efforts is to broaden to further efficient quantum chemical codes. For example, the simulation of very large chromophores can be made feasible with graphics processing unit accelerated electronic structure codes, as the inspiring work of Penfold (2017) recently showed for the direct‐dynamics variational multiconfigurational Gaussian method. Moreover, for the treatment of chromophores embedded in complex biological environments, SHARC will benefit from further development of interfaces to hybrid QM/MM methods. On the other extreme, small systems will profit from very accurate electronic structure methods with analytical gradients (Celani & Werner, 2003; MacLeod & Shiozaki, 2015). An entirely different possibility is offered by using machine learning potentials (Behler, 2017; Gastegger, Behler, & Marquetand, 2017; Hase, Valleau, Pyzer‐Knapp, & Aspuru‐Guzik, 2016; Ramakrishnan & von Lilienfeld, 2017) and extending them for the treatment of nonadiabatic dynamics.

## CONFLICT OF INTEREST

The authors have declared no conflicts of interest for this article.

## RELATED WIREs ARTICLES

https://doi.org/10.1002/wcms.1158

https://doi.org/10.1002/wcms.64
